# Involvement of the N Domain Residues E34, K35, and R38 in the Functionally Active Structure of Escherichia coli Lon Protease

**DOI:** 10.32607/actanaturae.11197

**Published:** 2020

**Authors:** A. G. Andrianova, A. M. Kudzhaev, V. A. Abrikosova, A. E. Gustchina, I. V. Smirnov, T. V. Rotanova

**Affiliations:** Shemyakin-Ovchinnikov Institute of Bioorganic Chemistry, Russian Academy of Sciences, Moscow, 117997 Russia; Macromolecular Crystallography Laboratory, NCI-Frederick, P.O. Box B, Frederick, MD 21702, USA

**Keywords:** cellular proteome quality control, AAA+ proteins, ATP-dependent proteolysis, LonA proteases, N domain

## Abstract

ATP-dependent Lon protease of *Escherichia coli *(*Ec*Lon), which belongs to the superfamily of AAA^+^ proteins, is a key component of the cellular proteome quality control system. It is responsible for the cleavage of mutant, damaged, and short-lived regulatory proteins that are potentially dangerous for the cell. *Ec*Lon functions as a homooligomer whose subunits contain a central characteristic AAA^+^ module, a C-terminal protease domain, and an N-terminal non-catalytic region composed of the actual N-terminal domain and the inserted α-helical domain. An analysis of the N domain crystal structure suggested a potential involvement of residues E34, K35, and R38 in the formation of stable and active *Ec*Lon. We prepared and studied a triple mutant LonEKR in which these residues were replaced with alanine. The introduced substitutions were shown to affect the conformational stability and nucleotide-induced intercenter allosteric interactions, as well as the formation of the proper protein binding site.

## INTRODUCTION

ATP-dependent Lon proteases (MEROPS: clan SJ, family S16) are key components of the cellular protein quality control system that ensures proteome homeostasis in all kingdoms of nature. Along with Lon and other ATP-dependent proteases, the protein quality control (PQC) system includes molecular chaperones that are responsible for correct protein folding, formation of protein assemblies, and prevention of aggregate accumulation in the cell. In turn, ATP-dependent proteases and multisubunit bifunctional complexes, proteasomes, degrade damaged, mutant, and short-lived regulatory proteins that are potentially dangerous for the cell [1-6].

**Fig. 1:**
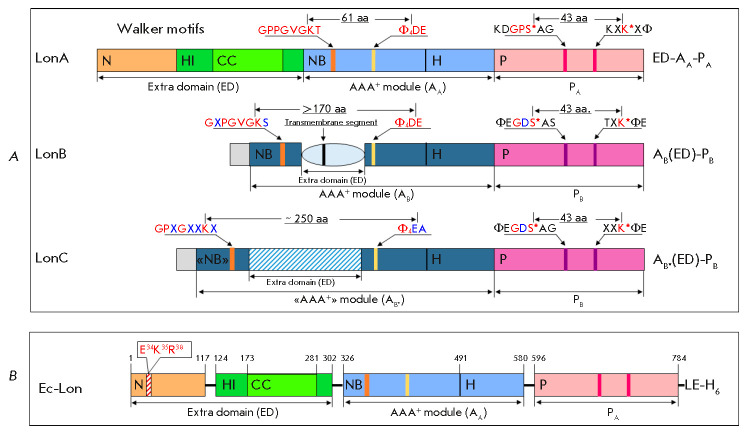
Domain organization of Lon proteases from different subfamilies (*A*) and domain boundaries in the subunit of *E. coli *Lon protease (*B*). (*A*) S* and K* – catalytic residues of the proteolytic active site; Φ – hydrophobic amino acid residue; X – any amino acid residue; **PA **and **PB **– A-type (pink) and B-type (purple) protease domains; **AA**, **AB**, and AB* – AAA^+^ modules of A-type (light blue), B-type (blue), and “degenerate” B*-type (blue), respectively; **NB **– nucleotide-binding domain; **H **– α-helical domain; **ED **– extra domains represented by the **N-**domain (brown) and inserted α-helical **HI(CC) **domain (green) with a coiled-coil region (light green) in LonA proteases, a transmembrane domain (light blue) in LonB, and an inserted domain (shaded) in LonC; aa – amino acid residue; amino acid substitutions in conserved fragments are highlighted in blue. (*B*) *E. coli *Lon protease subunit with a C-terminal 6His-tag; the N domain region comprising E34, K35, and R38 residues is shaded

Lon proteases are homooligomeric enzymes. Their subunits include the ATPase (AAA^+^) module formed by the nucleotide binding (NB) and α-helical (H) domains, the protease (P) domain that is a serine-lysine peptide hydrolase, and either the N-terminal or the inserted non-catalytic extra domain (ED) (*Fig. 1*) [7, 8].

Because Lon proteases, as well as other PQC proteases, contain the AAA^+^ module in their structure, they belong to the superfamily of AAA^+^ proteins (ATPases Associated with a variety of cellular Activities) that are abundant in nature and involved in important processes, such as DNA replication, transcription, cell division, intracellular transport, folding, proteolysis, etc. [9-12]. AAA^+^ proteases are highly selective enzymes. Their main features are coupling of proteolytic activity with ATP hydrolysis and processive hydrolysis of protein targets to form extremely low-molecular-weight products (5–15 amino acid (aa) residues) [13, 14, 15].

ATP-dependent proteases select their substrates from a variety of cellular proteins based on the pres ence of special structural elements: exposed hydrophobic protein regions or labels called degrons. Degrons are specific amino acid sequences located at the end or inside of a substrate polypeptide chain [16, 17, 18]. Protein called ubiquitin serves as a label of substrates for eukaryotic proteasomes [19, 20]. The processive mechanism of substrate hydrolysis by AAA^+^ proteases is implemented through a barrel-like quaternary structure of these enzymes. Their cylindrical oligomers use ATP energy for binding, denaturation, and translocation of protein substrates through the central pore, which is formed by stacked rings of ATPase modules and protease domains, to peptidase centers hidden within the enzyme oligomer [21, 22, 23].

To date, three subfamilies (A, B, and C; *Fig. 1A*) have been identified in the total pool of ATP-dependent Lon proteases in the MEROPS database. Differences in the environment of the catalytically active serine and lysine residues of proteolytic centers and the localization of extra domains controlling the ATPase component architecture serve as the basis for allocation of Lon enzymes into subfamilies [7, 8, 24]. Two types of proteolytic centers have been identified in the Lon protease family: the PA type located in the P domains of the enzymes of the largest LonA subfamily comprising bacterial and eukaryotic enzymes [7, 8, 24, 25], and the PB type detected in the enzymes of the archaeal LonB subfamily [8, 26] and a small bacterial subfamily, LonC (*Fig. 1A*) [27, 28].

The extra domain of LonA proteases is an extended N-terminal region that provides a distinctive feature of members of this subfamily. LonB and LonC proteases contain inserted extra domains located in their nucleotide-binding domains, between the Walker A and B motifs. A specific feature of the extra domain of LonB enzymes is its transmembrane segment. The extra domain of LonC proteases is characterized by being longer compared to that of the LonB extra domain and by degeneration of the ATPase function due to a replacement of some essential residues of the ATPase site (*Fig. 1A*). However, LonC proteases are also involved in the protein quality system because regulation of their proteolytic activity is mediated by their retained ability to bind nucleotides [27].

Members of the LonA subfamily have been explored most extensively. Their N-terminal region has a two-domain structure [21, 29]. In the LonA protease of *E. coli *(*Ec*Lon), this region includes 325 aa and is formed by the “true” N-terminal (M1–Y117) and α-helical-inserted HI(CC) (E124–P302) domains (*Fig. 1B*) [29, 30]. The former has a twisted β-sheet structure and is topologically similar to RNA-binding PUA domains [31, 32]. The latter domain is formed by eight α-helices. It includes a region with a specific coiled-coil (CC) conformation, and moreover it is highly similar to the H domain of its own AAA^+^ module, as well as to the α-helical domain of the first AAA^+^ module of chaperone disaggregases ClpB/Hsp104, which contains an inserted M domain with a CC conformation [30, 31, 33].

To date, a lot of evidence has been accumulated showing the role of the AAA^+^ module and protease domain in the functioning of LonA proteases. However, the functions of the N-terminal region of LonA proteases have not yet been fully characterized. According to published data, this region of the molecule is involved in the recognition and binding of substrate proteins [34-37]. Recently, the N-terminal region has been shown to participate in the formation of dodecameric structures from *E. coli *LonA protease hexamers [38, 39]. In addition, difference in the functions of the N and HI(CC) domains in the full-length *Ec*Lon protease has been revealed [40-45], confirming the two-domain organization of the enzyme’s N-terminal region. Results of various studies indicate a crucial role played by the N-terminal region of LonA proteases in maintaining their functionally active conformation. In this case, it remains unclear which fragments of the N domain are important for the structural organization and are involved in the stabilization of enzymes.

The aim of this study was to identify the N-terminal domain residues involved in the formation of a stable, functionally active structure of the *Ec*Lon protease (hereinafter referred to as Lon protease), perform site-directed mutagenesis of these residues in order to produce a mutant enzyme, and investigate the structural and enzymatic characteristics of the mutant compared to those of intact *Ec*Lon.

## EXPERIMENTAL

**Materials**

We used commercial reagents from Sigma, Bio-Rad, Thermo Scientific (USA), Fluka, Bachem (Switzerland), Boehringer Mannheim (Germany), Pharmacia (Sweden), Difco (England), Panreac (Spain), and Reakhim (Russia).

**Preparation of recombinant** *Ec* **Lon protease (Ec-Lon) and its mutant form, LonEKR**

Recombinant *Ec*Lon protease containing a hexahistidine fragment within the LEHHHHHH octapeptide at the C terminus of the protein (Ec-Lon) was produced according to a previously described procedure [40].

A triple mutant LonEKR was produced based on a megaprimer approach using the nucleotide sequence of Ec-Lon protease with the following primers: Lon_E34K35R38/AAA, T7 promoter, and f9 (5′-CCATCGCCGCTTCCAGACA AGCGATAGATGCTGCCCGCCCGACAAATAAGGGG-3′, 5′-TTAATACGACTCACTATAGGGGA-3′, and 5′-CGTTTACACCCGGCTCATCC-3′, respectively). The gene fragment was amplified in two stages using plasmid DNA pET28-Ec-*lon *as a template. At the first stage, Lon_E34K35R38/AAA and T7 promoter primers were used to prepare a PCR fragment that, together with the f9 primer, was used as a primer at the second stage. The produced DNA fragment of about 250 bp was cloned into the pET28-Ec-*lon *vector at the unique XbaI and HindIII restriction sites.

Cloned DNA sequencing and primer synthesis were performed by EVROGEN (www.evrogen.ru). Restriction and ligation procedures were performed according to the protocols of the enzyme’s manufacturers.

*E. coli *BL21(DE3) cells carrying the pET28-*lonEKR *plasmid were cultured in a LB medium with kanamycin at 37°C with vigorous stirring until OD_600_ reached 0.5, then the cell culture temperature was lowered to 25°C, and induction at 0.1/1 mM IPTG was performed for 3 h.

Ec-Lon and LonEKR were isolated and purified using Ni^2+^-chelate affinity chromatography (HisTrap FF column, 5 mL, GE Healthcare, USA) and anion exchange chromatography (HiTrap^TM^ Q FF column, 5 mL, GE Healthcare) according to the previously described procedure [40], followed by two-stage gel filtration on HiPrepTM 16/60 Sephacryl S-300 HR (120 mL, GE Healthcare) with the following buffers: 50 mM imidazole, pH 7.5, 0.5 M NaCl (stage 1) and 50 mM Tris-HCl, pH 7.5, 0.5 M NaCl (stage 2).

Protein concentrations were determined using the Bradford method [46].

The homogeneity of protein samples was tested electrophoretically [47] using a commercial set of markers (kDa): β-galactosidase (116.0), bovine serum albumin (66.2), ovalbumin (45.0), lactate dehydrogenase (35.0), restriction enzyme Bsp98I (25.0), β-lactalbumin (18.4), and lysozyme (14.4).

**Determination of the enzymatic properties of Ec-Lon and its triple mutant LonEKR**

*ATPase activity *was tested based on the kinetics of inorganic phosphate accumulation in the ATP hydrolysis reaction in 50 mM Tris-HCl buffer, pH 8.1, containing 200 mM NaCl, 2.5 mM ATP, 2.5 or 20 mM MgCl_2_, and 0.1–1.0 μM enzyme, with and without β-casein (1 mg/mL), at 37°C [48]. In the control experiment, the enzyme was replaced with the buffer. The initial reaction rates were determined using the OD value of a mixture of 200 μL of the reaction medium and 600 μL of the reagent (100 mM Zn(AcO)_2_, 15 mM (NH4)6Mo7O_2_4, 1% SDS, pH 4.5–5.0) at a wavelength of 350 nm (ε_350_ = 7,360 M^–1^ cm^–1^).

*Thioesterase activity*. Hydrolysis of a thiobenzyl ester of the N-protected tripeptide Suc-Phe-Leu-Phe-SBzl (PepTBE) was monitored spectrophotometrically at a wavelength of 324 nm using the OD value of 4-thiopyridone (ε324 = 16,500 M–1 cm–1) formed in the reaction between a hydrolysis product (benzyl thiolate, BzlS–) and 4,4′-dithiodipyridine (DTDP) [49]. PepTBE was hydrolyzed at 37°C in 50 mM Tris-HCl buffer, pH 8.1, containing 200 mM NaCl, 10% DMSO, 0.2 mM DTDP, 0.1 mM PepTBE, and 0.1–1.0 µM enzyme. When studying the influence of effectors, a nucleotide, up to 2.5 mM, and MgCl_2_, up to 20 mM, were added to the mixture.

*Proteolytic activity *of enzymes was tested electrophoretically [47]. The reaction was conducted at 37°C in 50 mM Tris-HCl buffer, pH 8.1, containing 200 mM NaCl, 20 µM β-casein, and 1 µM enzyme, with and without 5 mM Nu and 20 mM MgCl_2_. In the control experiment, the enzyme was replaced with the buffer. An aliquot of the reaction or control mixture was mixed with the lysis buffer (0.2 M Tris-HCl, pH 8.9, 4% SDS, 20% glycerol, 0.5 mM EDTA, 0.8% bromophenol blue, 3% β-mercaptoethanol) at a 3 : 1 ratio, boiled for 5 min, and applied to a 12% polyacrylamide gel for electrophoresis.

*The autolytic activity *of enzymes was tested electrophoretically [47] under conditions similar to those for determining the proteolytic activity, but in the absence of β-casein.

*Limited chymotrypsin proteolysis *of Ec-Lon protease and its triple mutant LonEKR was carried out at 30°C in 50 mM Tris-HCl buffer, pH 8.1, containing 300 mM NaCl, 11 μM enzyme, and 0.2 μM chymotrypsin, with and without *Ec*Lon protease effectors.

## RESULTS AND DISCUSSION

**Identification of** *Ec* **Lon protease N domain residues presumably involved in formation of the functionally active enzyme**

Previously, we have shown that the HI(CC)-inserted domain plays the key role in the correct binding of a protein substrate by the *Ec*Lon protease, efficient functioning of its ATPase and peptidase centers, implementation of intercenter allosteric interactions, and the processive mechanism of proteolysis [40-45]. In this case, the (E124–H172) and (M281–N302) fragments flanking the CC region were critically important for the interaction with a protein substrate and its hydrolysis [41, 42, 43].

We found [44] that the N-terminal domain ensures the conformational stability of the *Ec*Lon protease upon coupling of proteolysis with ATP hydrolysis, because a truncated enzyme (G107–K784) produced by the removal of the (M1–N106) fragment undergoes intensive autolysis, despite the preserved ability for processive proteolysis. In addition, the N-terminal domain residues R33, E34, and K35 were shown to be involved in the specific binding of *Ec*Lon substrates containing the so-called sul20-degron (a fragment of the cell division inhibitor SulA), which, in turn, affects the activities of ATPase and proteolytic centers [34].

**Fig. 2:**
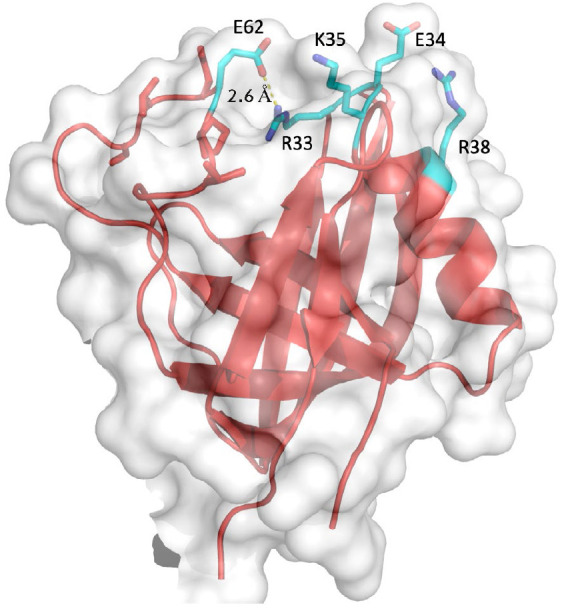
Cartoon representation of the *Ec*Lon N domain comprising residues 7–118, with side chains of the R33, E34, K35, R38, and E62 residues shown in sticks. The solvent accessible surface of the protein is shown in light gray

At the same time, the results of the X-ray diffraction analysis of the N-terminal region of *E. coli *LonA protease [31] suggest that the region containing residues R33, E34, K35, and R38 may be important for interdomain and/or intersubunit interactions in the enzyme. This region is located on the surface of the *Ec*Lon protease N domain (*Fig. 2*), and, therefore, these residues may be directly involved in both the interactions with the substrate and the interactions between the protomers within the *Ec*Lon oligomers. The suggestion about the involvement of this region in the active structure and functioning of *Ec*Lon can be verified by studying the properties of a mutant enzyme with substitutions of potentially significant residues.

However, *Fig. 2* shows that the R33 residue forms an ion pair with the E62 residue located at the end of an 18 aa surface loop. This interaction restricts the mobility of this loop and, thereby, maintains its conformation. Mutation of the R33 residue may impair the topology of the studied region. For this reason, in this study, we investigated an *Ec*Lon protease mutant (LonEKR) in which only three residues, namely E34, K35, and R38, were substituted with alanine.

**Preparation of the LonEKR triple mutant of *E. coli *Lon protease**

The LonEKR mutant containing the E34A, K35A, and R38A substitutions was produced using recombinant *Ec*Lon containing a hexahistidine fragment at the C-terminus of the protein (Ec-Lon) [40]. The intact enzyme and its triple mutant were isolated according to a scheme including affinity chromatography on Ni-Sepharose, ion-exchange chromatography on Q-Sepharose, and gel filtration on Sephacryl S-300. The ATPase, peptidase, proteolytic, and autolytic activities were determined for the intact and mutant enzymes. When studying ATP hydrolysis, the effects of excess magnesium ions and of the protein substrate were evaluated. The peptidase (substrate, Suc-Phe-Leu- Phe-SBzl (PepTBE)), proteolytic (model protein substrate, β-casein), and autolytic activities were tested with and without Lon protease effectors – nucleotides and magnesium ions.

**ATPase activity of the LonEKR mutant**

Previously, intact Ec-Lon protease was shown to exhibit maximum ATPase activity in the reaction medium at pH 8.0–8.2 and at 2.5 mM equimolar ATP and magnesium ion concentrations. An increased concentration of Mg^2+^ ions, which is typical of physiological conditions (20 mM), results in a decrease in the ATPase activity. A protein substrate can restore the rate of ATP hydrolysis to its optimal values [40, 43].

**Fig. 3:**
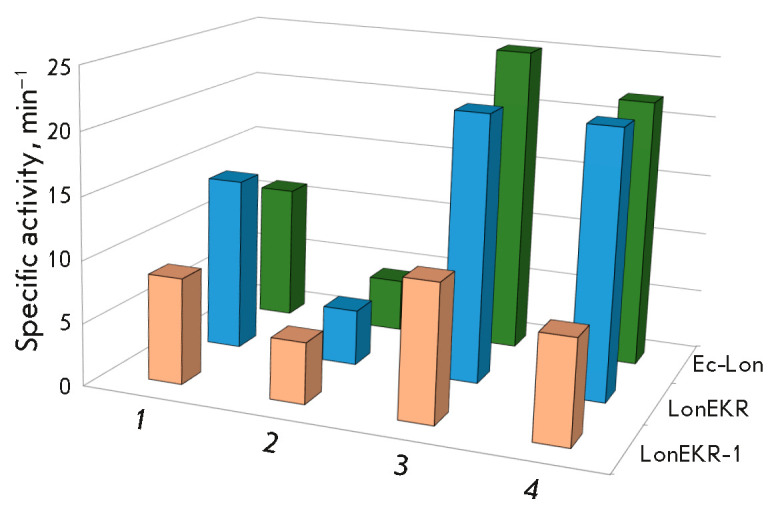
ATPase activity of intact Ec-Lon protease and its LonEKR and LonEKR-1 mutants. Experimental conditions: 50 mM Tris-HCl buffer, pH 8.1; 0.2 M NaCl; 37°C; concentrations: 2.5 mM ATP; 2.5 (*1*, *3*) or 20 mM (*3*, *4*) MgCl_2_; 0 (*1*, *2*) or 1.0 mg/mL (*3*, *4*) β-casein; 0.1–1.0 μM enzyme. The root-mean-square deviation R2 in the experiments was 0.98–1.00

The efficiency of ATP hydrolysis by the triple Ec- Lon protease mutant is close to that of the intact enzyme; in this case, the mutant retains its functional features, including inhibition by an excess of magnesium ions and subsequent activation of ATPase centers by β-casein (hereinafter referred to as casein). However, activation of the centers in the mutant in response to any interaction with casein is less effective than that in the intact Ec-Lon protease (*Fig. 3*), which may be due to weaker binding of the protein target caused by mutations of the E34, K35, and R38 residues.

In a separate experiment, producer strain cultivation conditions, in particular the induction condition, were shown to affect the efficiency of LonEKR ATPase centers. For the Ec-Lon protease and its modified forms, the optimal conditions were chosen as those reducing the crowding effect during expression of the target gene: fermentation was performed in the presence of 0.1 mM isopropyl-β-D-1-thiogalactopyranoside (IPTG) at a temperature of 25°C. As the inducer concentration increased to 1 mM, the baseline ATPase activity of an isolated mutant (LonEKR-1) decreased by 40% compared to that of the intact enzyme (*Fig. 3*). The efficiency of LonEKR-1 ATPase activity recovery upon interaction with a protein substrate was also noticeably lower than that of the intact Lon protease and LonEKR mutant (*Fig. 3*). This suggests that IPTG at a concentration of 1 mM adversely affects the folding of the Ec-Lon protease mutant, which is also confirmed by LonEKR-1 gel filtration experiments demonstrating broadening and tailing of the protein peak compared to Lon and LonEKR.

**Peptidase center activity of the LonEKR mutant**

**Fig. 4:**
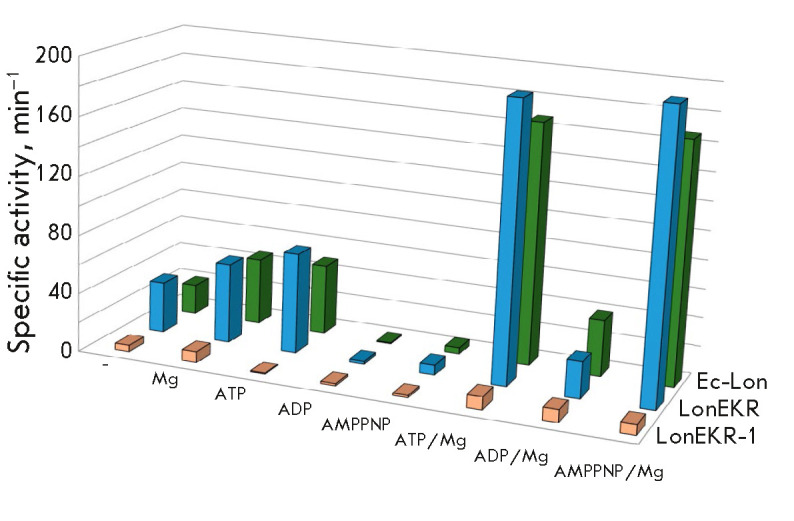
Peptidase activity of intact Ec-Lon protease and its LonEKR and LonEKR-1 mutants. Experimental conditions: 50 mM Tris-HCl buffer, pH 8.1; 0.2 M NaCl; 10% DMSO; 0.2 mM DTDP; 37°C; concentrations: 0.1 mM PepTBE; 2.5 mM nucleotides; 20 mM MgCl_2_; 0.1–1.0 μM enzyme. The root-mean-square deviation R2 in the experiments was 0.98–1.00

The efficiency of the peptidase centers of the intact Ec-Lon protease and its LonEKR mutant was assessed by the hydrolysis of a thiobenzyl ester of the N-protected tripeptide Suc-Phe-Leu-Phe-SBzl (PepTBE) [40]. During hydrolysis of the peptide substrate in the absence of nucleotide effectors, the LonEKR mutant was found to be more efficient (1.7-fold) than the intact Lon (*Fig. 4*). In this case, magnesium ions do not significantly activate the peptidase centers of both forms. Among free nucleotides, only ATP exhibits a weak but similarly efficient activating effect, whereas ADP and AMPPNP equally inhibit the peptidase activity, which indicates a similar affinity of nucleotides for the intact and mutant enzymes. The ATP/Mg and AMPPNP/Mg complexes exert the strongest activating effect on the peptidase sites of both Lon forms (*Fig. 4*). This indicates that the peptide hydrolase centers of the triple mutant act, in general, like centers of the intact enzyme.

These findings suggest that mutations in the E34, K35, and R38 residues of the *Ec*Lon N-terminal domain do not lead to significant changes in the functioning of enzyme peptidase centers. However, because ATP/Mg- and AMPPNP/Mg-based activation of the intact Lon noticeably exceeds that of the LonEKR form, it may be assumed that transmission of allosteric signals from the ATPase center to the peptidase center changes in the mutant, probably due to the differences in the efficiency of binding of Nu/Mg complexes.

It should be noted that the LonEKR-1 enzyme form produced upon expression of the mutant Lon protease gene in the presence of 1 mM IPTG exhibits a drastically decreased peptidase activity compared to that of the LonEKR mutant (*Fig. 4*). In the absence of effectors, hydrolysis of a low-molecular-weight substrate by LonEKR-1 is 8-fold slower than that by LonEKR, but the activating effect of magnesium ions remains. In contrast to the effect on LonEKR, any free nucleotides inhibit the peptidase activity of LonEKR-1 and their complexes with Mg^2+^ accelerate peptide hydrolysis only 2-fold, on average, which differs little from the effect of magnesium ions. Thus, as in the case of ATPase activity, these findings indicate that induction in the presence of 1 mM IPTG leads to significant conformational disruption in the enzyme structure, which affects its functional activity.

**Proteolytic activity and autolytic properties of the LonEKR mutant**

**Fig. 5:**
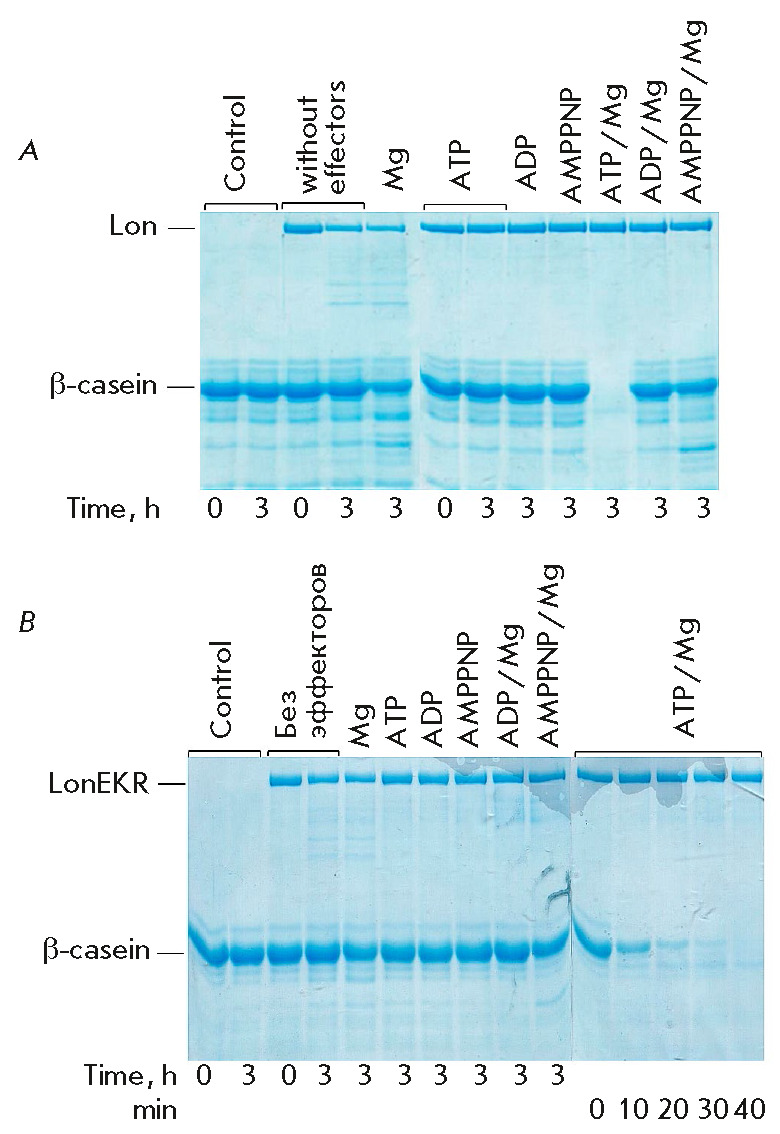
Hydrolysis of β-casein by Ec-Lon protease (*A*) and its LonEKR mutant (*B*) with and without effectors (electrophoresis in 12% PAAG). Experimental conditions: 50 mM Tris-HCl buffer, pH 8.1; 0.2 M NaCl; 37°C; concentrations: 20 μM β-casein; 5 mM nucleotides; 20 mM MgCl_2_; 1.0 μM enzyme

The proteolytic activity of Ec-Lon protease and its mutant was assessed by hydrolysis of β-casein (*Fig. 5*), similarly to refs. [40-45]. The LonEKR mutant retains the ability, characteristic of PQC enzymes, to hydrolyze a protein target via the processive mechanism (without releasing large intermediate products) upon coupling of proteolysis with ATP hydrolysis (*Fig. 5B*). This mechanism is implemented via the hexameric LonEKR structure, the formation of which was confirmed by gel filtration (data not shown). In the presence of the ATP/Mg complex, more than 50% of casein is degraded by the mutant in the first 10 min of reaction, which is comparable to the known efficiency of the ATP-dependent hydrolysis of this substrate by the native *Ec*Lon protease [43]. The intact enzyme is also characterized by an ability to degrade a protein substrate in the presence of the complex of a non-hydrolysable ATP analog, AMPPNP, with magnesium ions. In this case, the reaction products are high-molecular-weight fragments; i.e., proteolysis occurs by a non-processive mechanism and with low efficiency (*Fig. 5A*). Magnesium ions may also be considered separately as activators of non-processive hydrolysis of casein by Ec-Lon protease (*Fig. 5A*). In contrast to the intact enzyme, the proteolytic activity of the LonEKR triple mutant in the presence of both magnesium ions and the AMPPNP/ Mg complex proves to be almost absent over the same period of time (*Fig. 5B*). These results may reflect both a decreased efficiency in the binding of a protein substrate to LonEKR and disruption of allosteric interactions between the ATPase and proteolytic centers in the mutant enzyme.

**Fig. 6:**
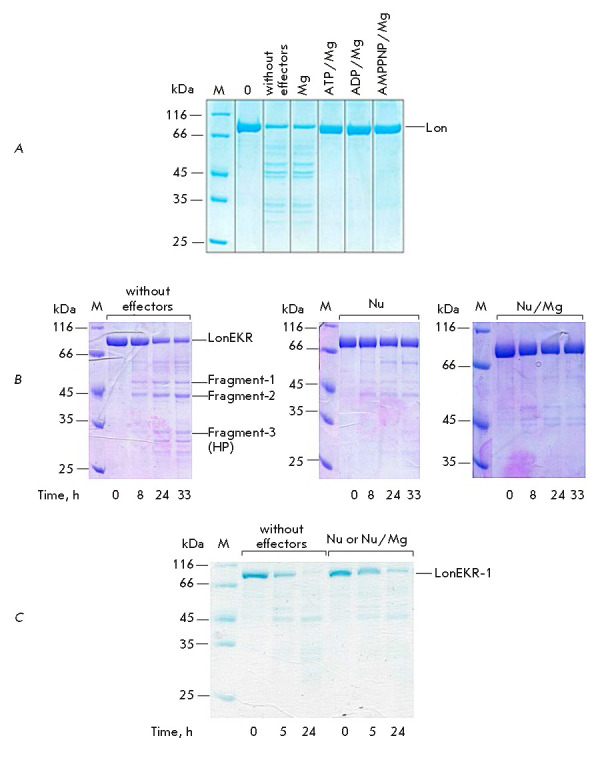
Autolytic properties of intact Ec-Lon protease (*A*) and its LonEKR (*B*) and LonEKR-1 (*C*) mutants. M – markers; Nu – nucleotide (ATP, ADP, or AMPPNP). Experimental conditions: 50 mM Tris-HCl buffer, pH 8.1; 0.2 M NaCl; 37°C; concentrations: 5 mM nucleotides; 20 mM MgCl_2_; 2.0 μM enzyme. (*A*) reaction time – 36 h; 0 – control (reaction time – 0 h)

As seen in *Fig. 5*, interaction between the enzyme and a protein substrate in the absence of effectors and in the presence of magnesium ions is accompanied by pronounced autolysis of the intact Lon protease and weak autolysis of the mutant. Investigation of the autolytic function of the native and mutant Lon forms in the absence of a target protein showed that the amounts of both enzymes significantly decreased over the experimental time interval (36 h for Lon and 33 h for LonEKR) (*Fig. 6 A, B*). In this case, autolysis of the intact Lon occurred only in the absence of nucleotide effectors while autolysis of the LonEKR mutant occurred under any conditions, but nucleotides and their complexes with magnesium ions significantly stabilized the mutant enzyme.

N-terminal sequencing revealed that stable LonEKR fragments were formed by autolysis of the enzyme at bonds located in the inserted HI(CC) domain (F138– E139 and M234–K235) and at the boundary between the NB and H domains (L490–S491) (*Fig. 1B* and *Fig. 8*). The products of autolysis at the F138–E139 and M234– K235 bonds are a 50 kDa Fragment-1 and a 44 kDa Fragment-2, respectively, (*Fig. 6B*). In these products, the C-terminal regions of the LonEKR sequence (presumably P domains) are probably also cleaved. Autolytic cleavage of the triple mutant at the L490–S491 bond leads to formation of a Fragment-3 (33 kDa) that includes H and P domains (*Fig. 6B* and *Fig. 8*).

**Fig. 7:**
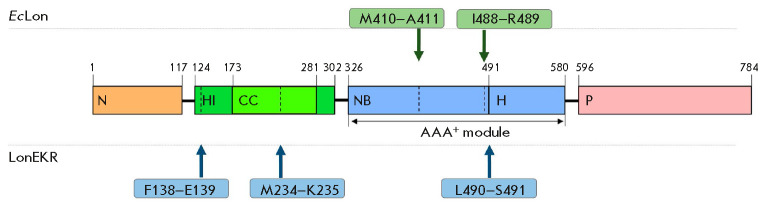
Location of autolysis sites in native *Ec*Lon protease and LonEKR mutant

**Fig. 8:**
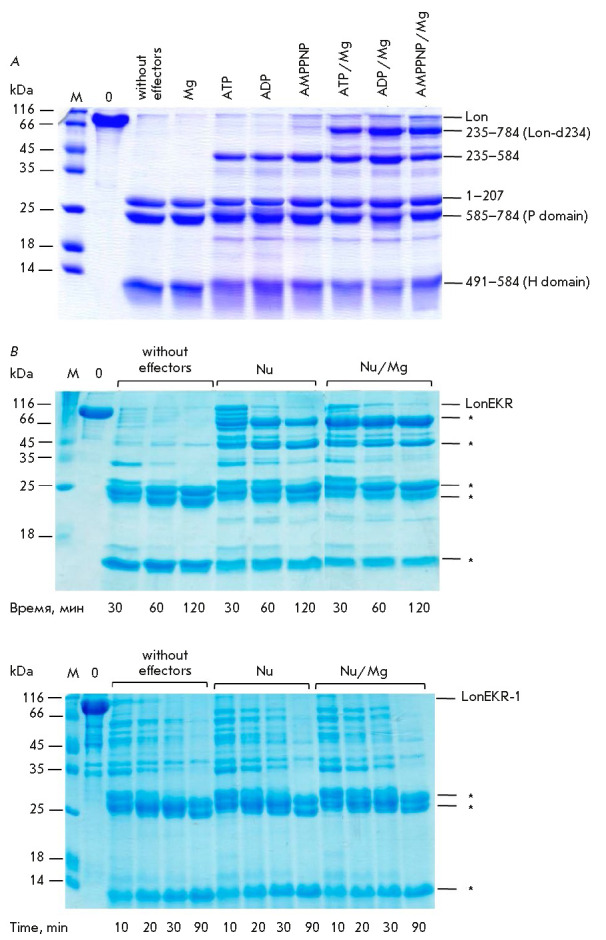
Chymotrypsinolysis of native *Ec*Lon protease (*A*) and LonEKR (*B*) and LonEKR-1 (*C*) mutants. M – markers; 0 – reaction mixture sample at initial time; Nu – nucleotide (ATP, ADP, or AMPPNP). * – Products of LonEKR and LonEKR-1 chymotrypsinolysis whose N-termini are not confirmed by sequence analysis. Experimental conditions: 50 mM Tris-HCl buffer, pH 8.1; 0.3 M NaCl; 30°C; concentrations: 11 μM Lon (LonEKR or LonEKR-1); 5 mM nucleotides; 20 mM MgCl_2_; 0.2 μM chymotrypsin. (*A*) reaction time – 2 h

Stable fragments of native Lon protease were formed during autolysis in the NB domain at the M410–A411 and I488–R489 bonds and only in the absence of nucleotide effectors [43] (*Fig. 8*). In the latter case, as in LonEKR, a 33 kDa fragment comprising α-helical and protease domains (HP) was formed. Thus, the autolysis results indicate a difference in the conformations of the intact Lon protease and its triple mutant LonEKR, as well as the potential effect of the introduced mutations on the efficiency of binding of Nu/Mg complexes.

Cleavage of the native enzyme at the M234–K235 bond located in the characteristic “long helix” of the CC region is also possible, but this degradation pattern occurs only upon limited chymotryptic proteolysis of Lon in the presence of nucleotides or Nu/Mg complexes [50]. Thus, it may be suggested that the M234–K235 and L490–S491 (or I488–R489) bonds are located in Lon subunit regions accessible to various proteases. However, cleavage of the F138–E139 bond in the N-terminal α-helix of the HI(CC) domain has not yet been found either in native Lon protease or in any of its modified forms.

Autolysis sites in the HI(CC) domain (aa 124–302), which are not typical of intact Lon protease, were previously found in three N-terminal domain-truncated enzymes in the presence of the ATP/Mg complex. For example, under these conditions, a Lon-d106 form lacking the first 106 aa undergoes intense cleavage of the A267–K268 bond located at the N-terminus of the last helix of the CC region [44]. Because Lon-d106 is the only truncated enzyme retaining an ability for ATP-dependent processive hydrolysis of a protein substrate, it was concluded that the Lon protease N domain is not involved in the processive proteolysis mechanism, but its presence ensures the conformational stability of the enzyme under classical conditions of its functioning [44]. A Lon-d172 form lacking the first 172 residues is also unstable in the presence of the ATP/Mg complex and undergoes autolysis of the D245–D246 bond (central part of the CC region) [43]. A Lon protease fragment, Lon-d234 (aa 235–784), produced by limited proteolysis also exhibits increased autolytic activity upon coupling with ATP hydrolysis: autolysis amounts to 50% just after 20 min, with the cleavage occurring immediately after the CC region at the A286–E287 bond [50].

Thus, the introduction of three mutations into the Lon protease N-terminal domain was shown to noticeably destabilize the enzyme and cause conformational changes permitting exposure to the environment of a natively hidden region comprising the N terminus of the α-helical HI(CC) domain.

It should be noted that these LonEKR features become even more evident when the mutant gene is induced under conditions not optimal for this enzyme (1 mM IPTG). The LonEKR-1 mutant produced in this way undergoes almost complete autolysis within a day, regardless of the presence of nucleotides or nucleotide-magnesium complexes in the reaction mixture (*Fig. 6C*).

**Fig. 9:**
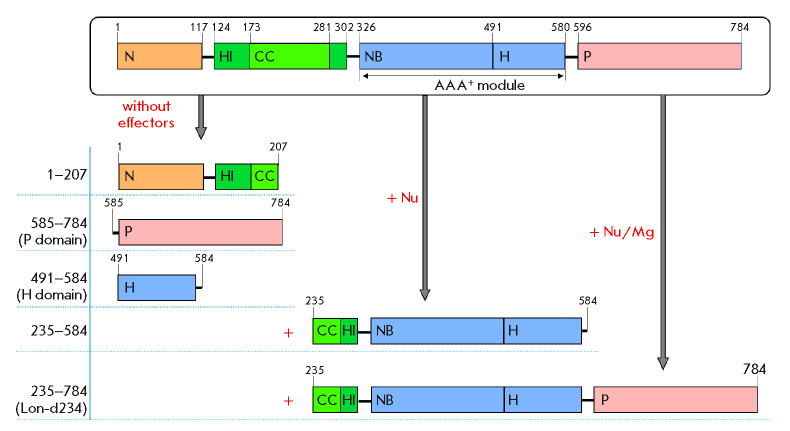
Structures of the products of *Ec*Lon limited proteolysis by chymotrypsin

To further characterize the conformational stability of Lon protease and its LonEKR mutant, we also used limited chymotryptic proteolysis. The result of chymotrypsinolysis of native Lon protease is effector-dependent [50]. In the absence of effectors, only the N-terminal fragment (1–207) and P and H domains are formed, whereas the presence of a nucleotide leads to stabilization of the central NB domain and, as a result, to formation of an additional fragment (235–584) involving the AAA^+^ module (326–584) and also a HI(CC) domain portion (235–302) with a linker (303–325) (*Fig. 1B* and *Fig. 8A*). The products of Lon protease chymotrypsinolysis are shown schematically in *Fig. 9*. The presence of nucleotide-magnesium complexes stabilizes the region between the ATPase module and the protease domain, which leads to formation of the fragment (235–784), referred to above as Lon-d234 (*Fig. 8A* and *Fig. 9*).

Limited chymotryptic proteolysis of the LonEKR form occurs in a similar way (*Figs. 8A and B*), and it may be assumed that the resulting fragments do not differ from the products of chymotrypsinolysis of the intact enzyme. However, in the case of the LonEKR-1 form produced with 1 mM IPTG, no stable NB domain-containing fragments of the sequence were detected either in the presence of nucleotides or in the presence of their complexes with magnesium ions (*Fig. 8B*). The chymotrypsinolysis results indicating that nucleotides and nucleotide-magnesium complexes do not stabilize the LonEKR-1 mutant structure are in full agreement with the autolysis data for this mutant. Therefore, induction of the *lonEKR *gene (1 mM IPTG) causes formation of an unstable conformation of the LonEKR-1 enzyme, which leads to its rapid autolytic cleavage under experimental conditions.

## CONCLUSION

We previously established that the N-terminal domain provides conformational stability to *Ec*Lon protease. In this study, on the basis of X-ray structural data, we proposed testing the role of residues E34, K35, and R38 of the N domain as amino acids involved in maintaining a stable structure of the functional enzyme through intersubunit and/or interdomain interactions. The replacement of these residues with alanine resulting in the triple LonEKR mutant was shown not to cause significant changes in the functioning of the ATPase and peptide hydrolase centers of the enzyme, but reduced binding of a protein substrate.

Like the native enzyme, the LonEKR mutant forms hexameric structures, but its ability to form dodecamers still remains unclear. Thus the LonEKR form retains the main property of ATP-dependent proteases – the ability to processively degrade a target protein when proteolysis is coupled with ATP hydrolysis, despite the detected disruption in intercenter allosteric interactions. However, in contrast to the intact enzyme, the LonEKR form is somewhat destabilized by the introduced substitutions because nucleotides and their complexes with magnesium ions, which are stabilizers of the Lon protease structure, are unable to completely prevent autolytic cleavage of the mutant.

It should be emphasized that gene induction and subsequent folding of the protein molecule play the key role in the formation of a stable structure of the functionally active Lon protease under crowding conditions. The LonEKR-1 form produced at a relatively high inducer concentration (1 mM IPTG) is not stabilized at all by nucleotides and exhibits an increased autolysis rate compared to the intact Lon and LonEKR form.

Therefore, this study has revealed that the N-terminal domain residues E34, K35, and R38 in the *Ec*Lon protease affect the formation of the correct binding site for a protein substrate, participate in the enzyme transformations caused by interaction with nucleotides, and maintain the conformational stability of the enzyme. Putative involvement of the studied residues in the formation of *Ec*Lon protease dodecameric forms may be a subject for future structural research into the properties of the LonEKR mutant.

## Abbreviations

AMPPNP: adenosine 5′-(β,γ-imido)triphosphate
DTDP: 4,4′-dithiodipyridine
Nu: nucleotide
PepTBE: Suc-Phe-Leu-Phe-SBzl
Suc: succinyl
OD: optical density
